# Supplementary material for: An Origami Paper-Based Device Printed with DNAzyme-Containing DNA Superstructures for Escherichia coli Detection
Source: Micromachines (Basel). 2019 Aug 12;10(8):531. doi: 10.3390/mi10080531 (PMC6722672; doi:10.3390/mi10080531)
Supplement: Supplementary file 1 [file micromachines-10-00531-s001.pdf]

An Origami Paper-Based Device Printed with DNAzyme-Containing DNA Superstructures for *Escherichia coli* Detection

Yating Sun [1], Yangyang Chang [1], Qiang Zhang [2] and Meng Liu [1,*]

1. School of Environmental Science and Technology, Key Laboratory of Industrial Ecology and Environmental Engineering (Ministry of Education), Dalian University of Technology, 116024 Dalian, China
2. School of Bioengineering, Dalian University of Technology, 116024 Dalian, China

* Correspondence: mliu@dlut.edu.cn

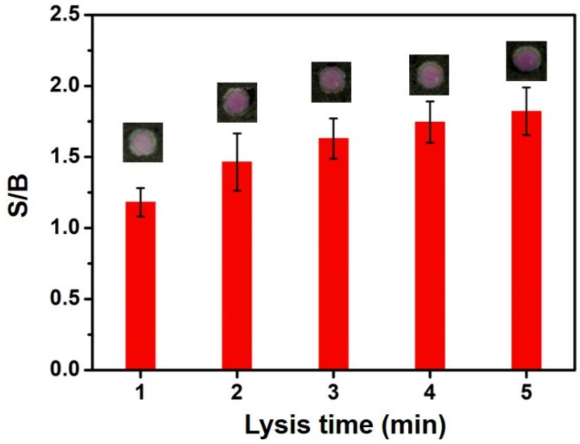

Figure S1. Determination of optimal lysing time on paper.
